# Development and internal validation of an age less-dependent frailty score in the cardiovascular health study

**DOI:** 10.3389/fmed.2025.1718015

**Published:** 2025-12-11

**Authors:** Aaron L. Troy, Brendon Choy, Huaying Dong, Julius M. Gardin, Calvin H. Hirsch, Angela S. Koh, William Kong, Kenneth J. Mukamal, Anne B. Newman, Michelle C. Odden, Michael Shu, Yang Song, Chenkai Wu, Jordan B. Strom

**Affiliations:** 1 Richard A. and Susan F. Smith Center for Outcomes Research in Cardiology, Beth Israel Deaconess Medical Center, Boston, MA, United States; 2 Division of Cardiology, Johns Hopkins School of Medicine, Baltimore, MD, United States; 3 Division of Cardiovascular Medicine, Beth Israel Deaconess Medical Center, Harvard Medical School, Boston, MA, United States; 4 Department of Medicine, Hackensack University Medical Center, Hackensack, NJ, United States; 5 Department of General Internal Medicine, University of California Davis Health, Sacramento, CA, United States; 6 National Heart Centre Singapore, Duke-National University of Singapore Medical School, Singapore, Singapore; 7 Department of Cardiology, National University Heart Centre, Singapore, Singapore; 8 Department of Epidemiology, University of Pittsburgh, Pittsburgh, PA, United States; 9 Department of Epidemiology and Population Health, Stanford University, Stanford, CA, United States; 10 University of Cincinnati College of Medicine, Cincinnati, OH, United States; 11 Global Health Research Center, Duke Kunshan University, Kunshan, China

**Keywords:** frailty, cardiovascular disease, biologic age, frailty score, aging

## Abstract

**Background:**

Frailty is a proxy for biologic aging that confers risk independently of chronologic age. Most frailty indices correlate strongly with chronologic age, making independent features of biologic aging challenging to identify.

**Methods:**

We aimed to create a novel Age Less-Dependent Frailty (AGELESS) Score less-associated with chronologic age than the Fried frailty phenotype. Among Cardiovascular Health Study participants with available echocardiographic data, we identified demographic, clinical, serologic, and echocardiographic variables more correlated with a continuous version of the Fried frailty phenotype than age, then used LASSO regression for variable selection. In a 25% leave-out sample, we internally validated the score's association with age-adjusted all-cause and cardiovascular mortality and compared model characteristics with the Fried frailty phenotype.

**Results:**

In 4,029 individuals (mean age 72 ± 5.0 years, 59.6% female), serum cystatin C, depression, diabetes, educational attainment, forced expiratory volume in 1 s, and income were more associated with frailty than age and selected for inclusion in the AGELESS Score. Adjusted for age, individuals in the highest vs. lowest quartiles of the AGELESS Score had a higher risk of all-cause (HR: 1.44, 95% CI: 1.17–1.79, *p* < 0.001) and CV death (HR: 1.64, 95% CI: 1.43–1.87, *p* = 0.002). The AGELESS Score was less correlated with age (AGELESS *r* = 0.23, 95% CI: 0.16–0.30; Fried *r* = 0.28, 95% CI: 0.21–0.34; *p*-value for comparison of correlations < 0.001) and more closely associated with all-cause and CV mortality within each age quartile than the Fried frailty phenotype.

**Conclusions:**

We derived and internally validated a novel frailty score that is less associated with chronologic age than existing indices and predicts mortality within age strata better than the existing reference standard for phenotypic frailty. This score could help identify high-risk patients with frailty across the age spectrum and may provide insights into mechanisms of biologic aging.

## Introduction

Frailty is a biologic syndrome of decreased reserve and resistance to stressors, resulting from cumulative declines across multiple physiologic systems (1). Frailty is present in at least 15% of community-dwelling older adults and represents an increasing global public health burden as the population ages (2, 3). Frailty is independently associated with increased risk of mortality and chronic illness, including cardiovascular disease, the leading global driver of mortality (4, 5). As a syndrome involving multiple biological changes, frailty is perhaps best represented by the Fried phenotype, consisting of a constellation of unintentional weight loss, self-reported exhaustion, weakness, slow walking speed, and low physical activity (1).

While the frailty syndrome, as a proxy for biologic aging, predominantly occurs in older adults, it can occur in individuals regardless of their chronologic age. This phenomenon is illustrated by many older individuals who are less frail than might be expected given their chronologic age and many younger individuals have severe frailty despite their age. At present, it is challenging to disaggregate the elements of biological aging that are unique vs. those that are a function of chronologic aging, as existing frailty metrics, including the Fried frailty phenotype and deficit-accumulation based indices, are comprised of variables that closely associated with chronologic age (6, 7). Understanding which features, augmented by echocardiographic measurements given the association between frailty and adverse cardiovascular outcomes, best associate with the frailty syndrome independent of chronologic age could deepen physiologic understanding of the frailty syndrome and its associations with other disease processes (8). A novel frailty score, constructed using these frailty-specific features, could help identify frailty-specific risk and potentially target candidates for interventions aimed at improving resilience, across the age-spectrum (9, 10).

As such, in this study, we evaluated participants in the National Heart, Lung, and Blood Institute (NHLBI)-funded Cardiovascular Health Study (CHS), to determine which clinical, serologic, echocardiographic, and sociodemographic variables associate differentially with frailty and chronologic age. Using these variables, we derived and internally validated a novel Age Less-Dependent Frailty (AGELESS) Score, designed to predict biologic aging, and evaluated its association with mortality and adverse cardiovascular outcomes.

## Methods

### Study population

CHS is a community-based, prospective, observational study that recruited adults >65 years from multiple communities across the United States. The first cohort enrolled predominantly White adults from 1989 to 1990 (*n* = 5,201) and the second cohort enrolled predominantly Black adults from 1992 to 1993 (*n* = 687) in an effort to increase diversity and generalizability (11, 12). All CHS participants who obtained a transthoracic echocardiogram were included in our study (*n* = 4,029). The CHS was approved by the Institutional Review Boards of all participating institutions, and this study was approved by the CHS Steering Committee and the Beth Israel Deaconess Medical Center Institutional Review Board with a waiver of informed consent.

#### Predictor variables

Possible predictors were determined using demographic, clinical, laboratory, and echocardiographic information obtained on CHS participants in 1989–90 and 1994–95 (for full list of predictors, see Table S1). Demographic, physical function, and Center for Epidemiologic Studies Depression (CES-D) scale data were obtained from the baseline examination, a 90-min in-home interview and clinical examination (11). The 10-item CES-D is a validated measure for self-reported depression symptoms over the previous week (1). Additional physiologic variables, including serologic, spirometric, and echocardiographic data were obtained during early study clinic visits, with all specific protocols and parameters previously described (11). M-mode, two-dimensional, and Doppler echocardiographic variables were measured using transthoracic echocardiograms obtained in 1989–90 (first cohort), or 1994–95 (second cohort), using standardized protocols (13). Most predictor variables were measured the same year as the baseline examination, and all were obtained within 2 years of enrollment.

#### Definition of frailty

Frailty can broadly be conceptualized either as a phenotypic syndrome or an accumulation of clinical deficits (14). This study utilized the Fried frailty phenotype, given its broad acceptance as a syndromic depiction of physical frailty (1, 15). This depiction was derived using the first CHS cohort then validated in both the second CHS cohort and additional large data sets, including the Women's Health and Aging Studies, the National Health and Aging Trends Study, and independently predicts risk of all-cause death, functional dependence, institutionalization, and cardiovascular disease among other patient-important clinical outcomes (1, 2, 5, 16). In the original reference standard Fried frailty phenotype, frailty is considered present when two or more components are abnormal including (1) unintentional weight loss >10 pounds or >5% of body weight in 1 year by direct measurement, (2) low grip strength measured by dynamometer, (3) self-reported exhaustion by two questions in the CES-D scale, (4) slow walking speed based on time to walk 15 feet, and low physical activity based on a weighted kilocalorie-per-week score in the baseline interview (1, 11). For this analysis, we used the Wu-Odden continuous version of the Fried frailty phenotype, developed to improve precision and avoid the ceiling effect (17). This continuous sum score was created by treating the five components of the Fried phenotype as continuous variables and using factor analysis to weigh each by its relative importance in measuring frailty (17). The score was validated for prediction of all-cause mortality and incident disability in both the CHS and the Health and Retirement studies (17).

#### Outcomes

Outcomes included all-cause mortality, cardiovascular-disease mortality, myocardial infarction, heart failure, and stroke or transient ischemic attack (TIA). Events, including cause of death, were rigorously adjudicated through 2015 by a clinical events adjudication committee, details of which have been previously published (18).

#### Statistical analysis

Participant characteristics of included patients are described using means and standard deviations (SDs) or medians and interquartile ranges (IQRs) for normal and non-normally distributed continuous variables and counts and percentages for categorical variables.

### Variable selection

Subjects were randomly assigned to derivation (75%) and validation (25%) cohorts. We subsequently standardized age, frailty, and continuous candidate variables to a mean of zero and standard deviation of one. Of 80 variables originally considered for inclusion, we selected variables with the least missingness (< 15%) within the 75% derivation sample, and remaining missing continuous values were imputed using the group median (19). Subsequently, we used simple linear regression to assess the association between each continuous predictor and both age and the continuous version of the Fried frailty phenotype. For categorical variables, the *F*-statistic derived from linear regression was used to identify the strength of association between each candidate predictor and age and frailty separately as outcomes. We identified 20 continuous variables and 10 categorical variables that were most strongly associated with frailty and less associated with age, based on ranking by the absolute differences in correlation coefficients or *F*-statistics for frailty vs. age (Table S2). Variables used in derivation of the Fried phenotype were not considered for inclusion, given their incorporation in the outcome variable.

### Model building

Split sample validation was used, deriving the score using a 75% random derivation sample and validating in a 25% leave-out validation sample. Using all identified candidate predictors in the 75% derivation sample, a multivariable linear regression model using LASSO regression for feature selection was used to estimate the association between candidate predictors and the standardized frailty metric. Candidate predictors were added to the model in a sequential and nested fashion, first starting with clinical, then laboratory, and subsequently echocardiographic variables due to the desire for parsimony and the increased clinical complexity of obtaining laboratory and echocardiographic information. For each nested model, Akaike information criteria (AICs), Bayesian information criteria, and log-likelihood ratios were used to determine model fit, with comparisons made across nested models using likelihood ratio tests. Clinical and laboratory variables were ultimately selected for inclusion in the model as they each improved fit by log likelihood ratio testing, while echocardiographic variables did not. The resulting model beta-coefficients and intercept were stored as the AGELESS Score, and this model was tested in the validation sample.

### Validation and outcomes

In the 25% leave-out validation sample, we assessed the association between the AGELESS Score and both the continuous Fried frailty phenotype and age, separately, using multivariable linear regression. We also evaluated the c-statistic the AGELESS score to predict frailty and the composite of frailty and pre-frailty using the original Fried phenotype definition in the validation cohort. Additionally, we computed adjusted *R*^2^ values for the AGELESS score and age against the continuous frailty outcome. The AGELESS Score was subsequently divided into quartiles and Cox proportional hazards models used to estimate the hazard ratios (HRs) and 95% CIs between AGELESS Score quartile and time to all outcomes, both with and without adjustment for age. Cumulative incidence functions were used to display the cumulative incidence of all outcomes by AGELESS Score quartile. Individuals were censored at either the date of last follow-up or end of study window, whichever occurred earlier. For non-death outcomes, we used cause-specific hazards techniques to account for the competing risk of death (20). Additionally, within each quartile of age, Cox proportional hazards models were used to estimate the association between each AGELESS Score quartile and the risk of all-cause and cardiovascular death, comparing model fit within each age stratum to the Fried frailty phenotype using the Akaike information criterion (AIC), with lower AIC indicating better fit. To improve etiologic understanding of how the AGELESS Score relates to the Fried frailty phenotype, we subsequently assessed correlations between each component of the Fried phenotype as well as the Fried phenotype as a whole and the variables included in the AGELESS Score. All analyses were performed using SAS v7.15 (SAS Institute Inc, Cary, NC) using a two-tailed *p*-value < 0.05 to declare significance.

## Results

### Participant characteristics

Of 5,888 total CHS participants, 4,029 individuals (68.4%; mean age 72.1 ± 5.0 years; 59.6% female; 83.5% White, 16.0% Black, 0.5% other race) received an echocardiogram in 1989–90 (*N* = 3,522) or 1994–95 (*N* = 507) and were included in the analysis (Figure S1). Most of the individuals in the cohort were retired (66.7% retired, 23.4% homemakers), married (67.9%), had completed at least high-school level education (28.7% high school graduation, 46.6% some college, graduate, or vocational school), and had a mean annual income between $16,000 and $24,999 per year. The mean Fried frailty phenotype was −0.1 ± 0.9. Included participants had a high burden of comorbidities including 45.1% with hypertension, 20.3% with chronic bronchitis, 18.6% with cancer, 15.0% with angina, and 13.8% with diabetes (Table 1 and Table S3).

**Table 1:** Participant characteristics^*^.

**Characteristics**	**Overall**	**Derivation (*n* = 3,021)**	**Validation (*n* = 1,008)**
**Demographics**			
Age (mean ± SD)	72.1 ± 5.0	72.1 ± 5.0	72.2 ± 5.1
Female sex	59.6%	60.0% (58.2%, 61.7%)	58.3% (55.2%, 61.4%)
Height, cm (mean ± SD)	165.0 ± 9.4	164.8 ± 9.4	165.5 ± 9.3
Weight, lbs (mean ± SD)	160.7 ± 31.2	160.7 ± 31.1	160.8 ± 31.4
**Race** ^†^			
White	83.5%	83.5% (82.2%, 84.9%)	83.4% (81.0%, 85.7%)
Black	16.0%	16.0% (14.7%, 17.3%)	16.1% (13.9%, 18.5%)
Other	0.5%	0.5% (0.3%, 0.8%)	0.5% (0.2%, 1.2%)
**Education** ^‡^			
Any lower-middle school	12.2%	12.5% (11.3%, 13.7%)	11.5% (9.5%, 13.6%)
Any high school	41.3%	41.4% (39.7%, 43.2%)	40.7% (37.7%, 43.8%)
Any vocational school	8.7%	8.6% (7.6%, 9.7%)	8.9% (7.2%, 10.8%)
Any college	26.6%	26.6% (25.1%, 28.2%)	26.4% (23.7%, 29.2%)
Any graduate or professional	11.3%	10.8% (9.8%, 12.0%)	12.5% (10.6%, 14.8%)
Household income^§^ (median)	$16,000–$24,999	$16,000–$24,999	$16,000–$24,999
**Clinical history** ^||^			
Angina	15.0%	15.2% (13.9%, 16.5%)	14.4% (12.3%, 16.7%)
Bronchitis	20.3%	20.6% (19.2%, 22.1%)	19.2% (16.8%, 21.8%)
Cancer	18.6%	18.8% (17.4%, 20.2%)	18.2% (15.8%, 20.7%)
Diabetes	13.8%	13.6% (12.4%, 14.9%)	14.4% (12.3%, 16.8%)
eGFR^¶^ (mean ± SD)	74.7 ± 16.7	74.8 ± 16.9	74.4 ± 16.2
Emphysema	3.1%	3.1% (2.5%, 3.8%)	3.2% (2.2%, 4.5%)
Heart failure	3.1%	3.1% (2.5%, 3.8%)	3.0% (2.0%, 4.2%)
Hypertension	45.1%	44.9% (43.1%, 46.7%)	45.6% (42.5%, 48.8%)
Myocardial infarction	7.7%	7.6% (6.7%, 8.7%)	7.9% (6.3%, 9.8%)
Tobacco use	52.8%	52.5% (49.6%, 55.4%)	44.8% (48.6%, 59.0%)
Stroke	3.0%	3.1% (2.6%, 3.8%)	2.6% (1.7%, 3.8%)
Transient ischemic attack	2.4%	2.4% (1.9%, 3.0%)	2.5% (1.6%, 3.6%)
Frailty^**^ (mean ± SD)	−0.1 ± 0.9	−0.1 ± 0.9	−0.2 ± 1.0

^*^Demographic and clinical data obtained at during the Cardiovascular Health Study baseline examination which consisted of a home interview/form and a clinic examination and took place in 1989–90 for cohort 1 and 1992–93 for cohort 2.

^†^Race was determined by subject self-report, with options being White, Black, American Indian/Alaskan native, Asian/Pacific Islander, and other: specify, and was included in the study to inform generalizability.

^‡^We grouped educational attainment into five groups based on highest level of education, any lower-middle school, any high school, any vocational school, any college, and any graduate or professional school.

^§^Annual household income, self-reported using ranges, with options including < $5,000, $5,000–7,999, $8,000–11,999, $12,000–15,999, $16,000–24,999, $25,000–34,999, $35,000–49,999, and over $50,000, and has not been adjusted for inflation.

^||^Clinical history questions were obtained via self-report, with the question, “has a doctor ever told you that you had…” or “have you been told by a doctor that you currently have any of the following conditions…”?

^¶^Estimated glomerular filtration rate (eGFR), reported in ml/min/1.73 m^2^ of body surface area, was calculated using the CKD-Epi equation using creatinine and cystatin c.

^**^Frailty is measured using the continuous frailty score created by Wu, et al. based on the Fried frailty phenotype, comprised of gait speed, grip strength, exhaustion, physical activity, and unintentional weight loss, with a population mean of 0.

### Univariable analysis

The dataset was randomly split into a 75% derivation cohort (*N* = 3,021) and a 25% validation cohort (*N* = 1,008). No significant differences in participant characteristics between derivation and validation cohorts were observed, aside from a 0.7 cm difference in height (derivation 164.8 ± 9.4 cm vs. validation 165.5 ± 9.3 cm, *p* = 0.04; Table 1). Of continuous measures evaluated, depression had the largest difference in correlation between frailty and age, followed by serum glucose and C-reactive protein (Table S2). Income and FEV1 were more negatively correlated with frailty than age whereas cystatin C was more positively correlated with age than frailty.

Among categorical variables tested, diabetes had the largest difference in association between frailty and age, followed by deep vein thrombosis, male sex, hypertension, stroke, heart failure, and educational attainment, all which had stronger positive correlations with frailty than age (Table S2).

### Creation of the AGELESS score

In the sequential, nested model creation process, clinical variables improved prediction [likelihood ratio (LR): 217.8, *p* < 0.001] as did serologic variables (LR: 70.6, *p* < 0.001), but echocardiographic variables did not (LR: 11.1, *p* = 0.35) and were therefore not included in the AGELESS Score. Of the clinical and serologic candidate predictors, LASSO regression selected depression (β = 0.046, 95% CI: 0.039–0.053, *p* < 0.001), cystatin C (β = 0.585, 95% CI: 0.446–0.724, *p* < 0.001), diabetes (β = −0.279, CI: −0.372 to −0.186, *p* < 0.001), income (β = −0.058, CI: −0.077 to −0.039, *p* < 0.001), FEV1 (β = −0.266, CI: −0.315 to −0.216, *p* < 0.001), and educational attainment (Table 2) for inclusion. The AGELESS Score, constructed using these variables, was moderately associated with frailty (r_frailty_ = 0.43, 95% CI: 0.37–0.49, *p* < 0.001), and was less associated with age than the Fried frailty score (*r* = 0.23, 95% CI: 0.16–0.30 for AGELESS Score vs. *r* = 0.28, 95% CI: 0.21–0.34 for the Fried frailty score, *p* < 0.001 for comparison of correlations). The adjusted *R*^2^ between frailty and the AGELESS score was 0.11 vs. 0.07 between frailty and age in the validation cohort. The AGELESS score was moderately predictive of both frailty (validation c-statistic = 0.697) and the composite of frailty and pre-frailty (validation c-statistic = 0.655) by the original Fried definition. CHS Site was not independently related to frailty in either derivation (*p* = 0.21) or validation (*p* = 0.83) cohort.

**Table 2:** AGELESS score variables.

**Continuous variable**	**Coefficient to normalized frailty**	**Coefficient to normalized age**	**Difference of absolute coefficients**	**β-coefficient**
Depression^*^	0.28	0.05	0.24	0.046 (0.039, 0.053)
Income^†^	−0.23	−0.11	0.12	−0.058 (−0.077, −0.039)
FEV1^‡^	−0.27	−0.21	0.06	−0.266 (−0.315, −0.216)
Cystatin C^§^	0.21	0.20	0.01	0.585 (0.446, 0.724)
**Categorical variable**	**F value for frailty**	**F value for age**	**Difference of F values**	β**-coefficient**
Diabetes^||^	43.40	1.67	41.73	−0.279 (−0.372, −0.186)
Education^¶^	17.72	9.13	8.59	
Any high school				0.242 (0.102, 0.382)
Any vocational school				0.016 (−0.091, 0.124)
Any college				−0.031 (−0.168, 0.107)
Any graduate or professional school				0.026 (−0.081, 0.133)
**Intercept**				
				0.129 (−0.115, 0.372)

^*^Measured using the Center for Epidemiologic Studies Depression (CES-D) Scale, which has a range of 0–60 based on symptoms experienced in the past week, with higher scores indicating more severe depression.

^†^Annual household income, self-reported using ranges, with options including < $5,000, $5,000–7,999, $8,000–11,999, $12,000–15,999, $16,000–24,999, $25,000–34,999, $35,000–49,999, and over $50,000, and has not been adjusted for inflation.

^‡^FEV1 was measured using a water-sealed spirometer.

^§^Serum Cystatin C was measured at the baseline examination and reported in mg/dl.

^||^Diabetes status was obtained by self-report, based on the question “have you been told by a doctor that you currently have diabetes”?

^¶^We grouped educational attainment into five groups based on highest level of education, any lower-middle school, any high school, any vocational school, any college, and any graduate or professional school.

### Death outcomes

**Figure 1:**
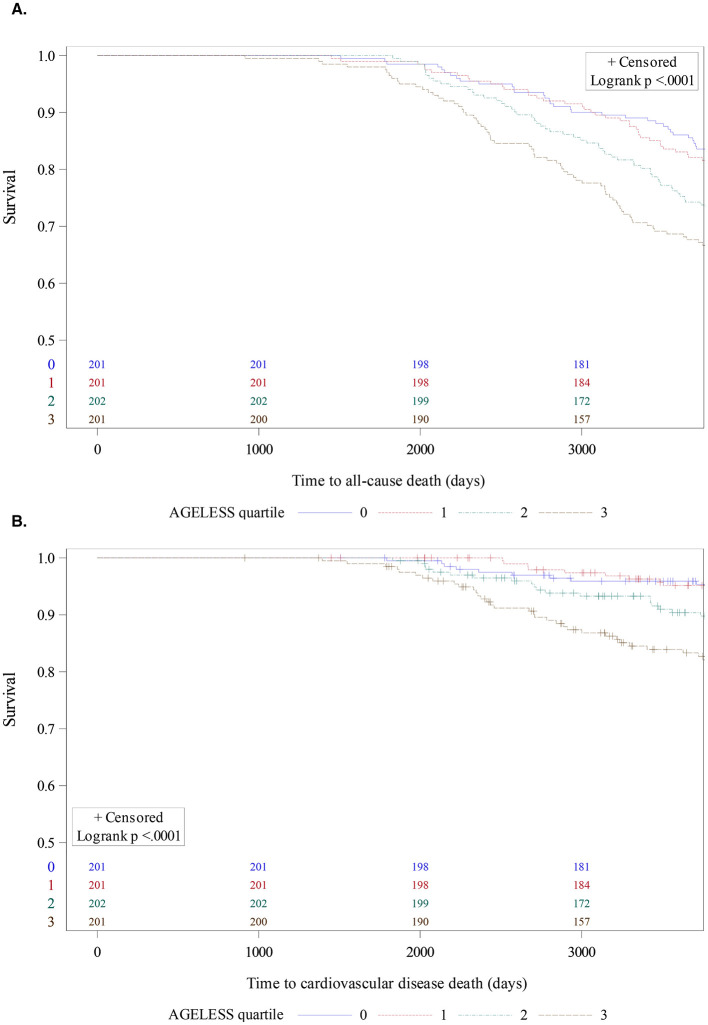
Ageless score and mortality. **(A)** All-cause death. **(B)** Cardiovascular disease death. AGELESS score reported by quartile, from lowest (0) to highest (3).

In the 25% validation cohort (*n* = 1,007), a total of 624 individuals died over a median of 14.9 (IQR: 10.3–20.5) years of follow-up. Compared to quartile 1 (AGELESS Score: < −0.18), both quartile 4 (AGELESS Score > 0.18) and quartile 3 (AGELESS Score: −0.02 to 0.18) were associated with an increased risk of all-cause death (quartile 4 vs. 1, HR: 1.72, 95% CI: 1.40, 2.13, *p* < 0.001; quartile 3 vs. 1, HR: 1.36 [95% CI: 1.10–1.68] *p* < 0.001; Table 3; Figure 1A). Compared to quartile 1, quartile 4 of the AGELESS Score was associated with an increased risk of cardiovascular death (quartiles 4 vs. 1, HR: 2.14, 95% CI: 1.50–3.04, *p* < 0.001; Table 3; Figure 1B).

**Table 3:** AGELESS score and mortality with and without adjusting for age.

**Death, adjusted for age**	**HR (95% CI)**	***p*-Value**
AGELESS quartile 2 vs. 1^*^	0.99 (0.80–1.23)	0.93
AGELESS quartile 3 vs. 1	1.19 (0.96–1.47)	0.11
AGELESS quartile 4 vs. 1	1.45 (1.17–1.79)	< 0.001
**Death, unadjusted**		
AGELESS quartile 2 vs. 1	0.98 (0.79–1.21)	0.86
AGELESS quartile 3 vs. 1	1.36 (1.10–1.68)	< 0.01
AGELESS quartile 4 vs. 1	1.72 (1.40–2.13)	< 0.001
**Cardiovascular death, adjusted**		
AGELESS quartile 2 vs. 1	0.87 (0.59–1.29)	0.46
AGELESS quartile 3 vs. 1	1.10 (0.75–1.60)	0.65
AGELESS quartile 4 vs. 1	1.79 (1.25–2.56)	< 0.01
**Cardiovascular death, unadjusted**		
AGELESS quartile 2 vs. 1	0.87 (0.59–1.28)	0.47
AGELESS quartile 3 vs. 1	1.25 (0.85–1.82)	0.25
AGELESS quartile 4 vs. 1	2.14 (1.51–3.04)	< 0.001

^*^AGELESS Score reported by quartile, from lowest (0) to highest (3).

After adjusting for age, quartile 4 of the AGELESS Score remained associated with all-cause (quartiles 4 vs. 1, age-adjusted HR: 1.45, 95% CI: 1.17–1.79, *p* < 0.001) and cardiovascular death (quartiles 4 vs. 1, age-adjusted HR: 1.79, 95% CI: 1.25–2.56, *p* = < 0.01; Table 3). Within each quartile of age, quartile 4 of the AGELESS Score was associated both all-cause and cardiovascular mortality (Table S4) and demonstrated improved model fit compared to the Fried frailty score for both all-cause death (AIC: 6,353.38 vs. 7,250.08) and cardiovascular death (AIC: 2,116.27 vs. 2,455.83).

### Non-death outcomes

After adjusting for age, the AGELESS Score was associated with some, but not all, non-death outcomes (Figure S2, Table S5). Specifically, quartile 3 of the AGELESS Score was associated with an increased risk of stroke or TIA before (quartile 3 vs. 1, HR: 1.78, 95% CI: 1.13–2.79, *p* = 0.01) and after adjusting for age (quartile 3 vs. 1, HR: 1.78, 95% CI: 1.13–2.79, *p* = 0.01). Within the second youngest age quartile (age 68–71), quartile 3 was also associated with an increased risk of stroke or TIA (quartile 3 vs. 1, HR: 3.71, 95% CI: 1.03–13.37, *p* = 0.045). Quartile 3 of the AGELESS Score was associated with incident heart failure on unadjusted analyses (HR: 1.42, 95% CI: 1.02–1.99, *p* = 0.04), but not after age adjustment (age-adjusted HR: 1.38, 95% CI: 0.99–1.92, *p* = 0.06). The AGELESS Score was not associated with an increased risk of incident myocardial infarction, regardless of age adjustment (all *p* > 0.05).

### Correlation of the AGELESS score and frailty domains

Correlations between the AGELESS Score and its components with the specific domains of frailty were assessed for etiologic purposes (Table S6). The AGELESS Score was most associated with increased exhaustion (*r* = 0.39, 95% CI: 0.33–0.45, *p* < 0.001), decreased walking speed (*r* = −0.36, 95% CI: −0.42 to −0.30, *p* < 0.001), and decreased grip strength (*r* = −0.35, 95% CI: −0.41 to −0.28, *p* < 0.001). Of AGELESS Score components, depression was most correlated with exhaustion (*r* = 0.41, 95% CI: 0.36–0.46, *p* < 0.001), cystatin C (*r* = −0.18, 95% CI: −0.25 to −0.12, *p* < 0.001) and diabetes (*r* = −0.08, 95% CI: −0.14 to −0.02, *p* = 0.01) were most correlated with decreased walking speed, FEV1 was most correlated with increased grip strength (*r* = 0.50, 95% CI: 0.44–0.54, *p* < 0.001), and educational attainment was most correlated with increased physical activity (*r* = 0.19, 95% CI: 0.13–0.25, *p* < 0.001).

## Discussion

In this study of 4,029 participants in CHS, we created and internally validated a novel frailty score that predicts risk of all-cause and cardiovascular disease mortality independent of chronologic age, performing better than the reference standard Fried frailty phenotype to identify outcome risks within age strata. This AGELESS Score includes depression, diabetes, cystatin C, FEV1, income, and educational attainment—all more strongly associated with frailty than chronologic age. This score may yield insights into the processes underlying biologic aging and could be used in clinical practice to better identify individuals who are frailer than expected for their age or who may benefit from early frailty-directed interventions (9).

To our knowledge, our study is the first to design a frailty score that is less associated with chronologic age than existing indices. Previous studies have used a wide-range of modalities to estimate biologic age, with the most prominent being DNA-methylation-based epigenetic clocks, proteomics, transcriptomics, immunomics, metabolomics, and deep learning models (21–24). Although these approaches have effectively captured clinically meaningful biologic age in the research setting, these approaches each have limitations. DNA-methylation and omics approaches lack gold-standard assays, are sensitive to lab and collection conditions, and are expensive (25). Most deep learning models require computational power and complete, specific, inputs. AGELESS and other clinical indices, by contrast, require minimal additional cost, time, and clinical sample collection. Moreover, frailty predicts survival better than DNA methylation, but has yet to be compared to most other proposed biologic age estimation modalities (26, 27). One study by Zhong et al. (28) estimated biologic age using clinically available markers and similarly found that biologic age was more associated with frailty and mortality than chronologic age with FEV1 and renal function found to be markers of biologic age. However, this study was conducted in 2,844 Chinese Singaporean subjects and used computationally intensive approaches for their final biologic age models (28). The AGELESS Score was constructed using a larger population more generalizable to US patients. Finally, the Targeting Aging with Metformin (TAME) Biomarkers Workgroup identified a set of eight biomarkers of aging for incorporation in geroscience clinical trials, two of them being A1c and cystatin C—components of the AGELESS Score (29). Although this study identified the important role of biomarkers in evaluation of biologic aging, it did not assess their relation with frailty.

From an explanatory perspective, the six markers of frailty selected for the AGELESS Score yield insight into the processes that drive frailty independent of age. The first, depression, has an established bidirectional relationship with frailty. Depression is associated with decreased physical activity, exhaustion, malnutrition, and psychomotor slowing—all known drivers of frailty—while frailty can promote functional and physical dependence, thereby increasing depression risk (25, 30). The second, diabetes, has a similar bidirectional relationship with frailty, as extremes of both hyperglycemia and hypoglycemia increase risk of developing frailty, and insulin resistance itself increases risk of sarcopenia (31–33). The third, cystatin C, both represents a muscle-mass independent marker of renal function and has been associated with frailty independent of estimated glomerular filtration rate (25, 34–36). The selection of cystatin C itself, rather than glomerular filtration rate measured using cystatin C, for final inclusion in the model may suggest these that cystatin C's pro-inflammatory and immune-mediating properties and its role as an inhibitor of the cysteine proteases which support protein turnover may underly its association with frailty above and beyond its association with glomerular filtration rate (35, 37). The fourth, the volume an individual can forcibly expel during the first second after inhalation—FEV1, has a multifaceted association with frailty (25). On one hand, FEV1 is a marker of pulmonary obstruction, inflammation, and endothelial dysfunction, which can limit physical activity and thereby worsen frailty (38, 39). On the other, decreased FEV1 could indicate decreased diaphragmatic strength from sarcopenia, as suggested by its association with grip strength in our study (39). Notably, depression, cystatin C, and FEV1 have been previously associated with frailty in a prior study of physiologic determinants of frailty independent of multimorbidity (52). The fifth, low income, has an established independent relationship with frailty mediated by social determinants of health, such as detrimental home and neighborhood environments, limited economic resources, and psychosocial factors including social isolation and perceived control (40). The sixth and final marker, educational attainment, has been found in prior studies to be inversely associated with frailty (41). However, in the current study, we found that higher educational attainment was associated with increased frailty in both unadjusted analysis and in our LASSO regression model. Reasons for this surprising and somewhat contradictory finding are unclear. It is possible that educational attainment is a marker for other socioeconomic factors associated with frailty, such as polypharmacy and occupational sedentary behavior (42). Further research exploring the associations between education, income, and frailty are warranted, as these social determinants are context-dependent and can impact frailty status through multiple mechanistic pathways (43).

Echocardiographic variables notably did not merit inclusion in the AGELESS Score, as they did not improve the model's predictive ability above clinical and serologic markers. This was also surprising, as cardiovascular imaging data, specifically, echocardiography and electrocardiography data, have shown promise as potential windows into cardiac age, which may closely approximate biologic age (21, 44). In a recent study, deep learning of echocardiographic images was able to identify predicted cardiac age, with discrepancies between predicted and actual chronologic age predicting increased risk of adverse outcomes (21, 44). Other cardiovascular parameters have been associated with increased frailty in specific settings, such as time-to-peak heart rate during gait assessment—possibly mediated by decreased muscular and metabolic reserve, and QRS duration in chronic hemodialysis patients—possibly mediated by myocardial fibrosis (45–47). In this context, there are several possible reasons echocardiographic data did not improve the AGELESS Score's predictive ability. First, it is possible that clinical and serologic data may capture similar information on an individual's frailty risk as echocardiography, as echocardiographic parameters are closely associated with clinical conditions and outcomes (48). Second, it is possible that radiomic features identified by deep learning may more closely associate with frailty and cardiac age than traditional echocardiographic measurements. Third, the high degree of missingness in echocardiographic variables (>15%), perhaps owing to limitations of echocardiography at the time CHS echocardiograms were performed, may have limited the impact of inclusion of these variables in the derivation model (49). More studies are necessary to explore the independent role of cardiac imaging data in identifying unique features of senescence.

The AGELESS Score is a unique tool that could, after external validation, be used to improve health outcomes for adults with frailty across the age spectrum. Firstly, having demonstrated improved prediction of mortality within age strata compared with the Fried phenotype, the AGELESS Score could be used in patients with suspected frailty out of proportion to their age to prompt evidence-based interventions such as physical rehabilitation, nutritional supplementation, cognitive training, comprehensive geriatric assessment and, in the future, pharmacologic interventions currently under investigation (9). Secondly, the score could be used in this patient population to help support risk/benefit discussions about medical procedures and interventions and identify ways to maximize benefit and minimize risk (5, 50). Finally, the score could identify patient and population-level targets for frailty prevention and improvement, such as mental health treatment for depression, diet and medication adjustment for improved glycemic control, inhalers and smoking cessation for pulmonary disease, and connection to social resources to support health-related social needs for low-income individuals.

While the CHS population was large, multicenter, and enrolling a community-dwelling older aged cohort, there are several limitations worth noting. First, causality should not be inferred using the existing methods. Second, there were varying degrees of missingness among variables in the CHS, which limited the information available for model building. Third, variables not collected as part of CHS may nevertheless associate with biologic age and thus variables included in the AGELESS Score should not be perceived as exhaustive. Fourth, there is no established gold standard for measurement of biologic age, so we used the Fried frailty phenotype for index creation and age-independent mortality for validation. Fifth, while the AGELESS score was less correlated with age than the Fried frailty score, both scores were only weakly associated with age and this difference, while statistically significant, should not connote that the AGELESS score is age-independent. Sixth, while split-sample validation was chosen to test the model in a leave-out sample, we acknowledge that there may be inefficiencies in this this approach compared to bootstrapping or cross-validation (51). Moreover, there may be benefits to using multiple imputation over median imputation, though it remains uncertain if this would substantially change results given the non-biased sampling in CHS (19). Accordingly, these approaches and the performance of the AGELESS score should be tested and results re-calibrated accordingly and if necessary in an external cohort. And finally, external validation with clinical performance testing will be necessary to confirm these findings and their generalizability before broad clinical use. Specifically, as CHS is predominantly a biracial cohort, the generalizability to other racial groups is less clear at present and, as CHS includes adults aged 65 and above, future studies would be needed to extend these findings to a younger cohort.

## Conclusions

Using data from CHS, we created and internally validated a novel frailty score, the AGELESS Score, to identify biologic aging independent of chronologic age. This score, comprised of information on depression, diabetes, cystatin C, FEV1, income, and educational attainment, performed better than the reference standard Fried frailty phenotype to identify outcome risks within age strata. This novel score identifies factors uniquely associated with biologic aging and could improve health outcomes in individuals frailer than expected for their age by enabling refined risk assessment and precise targeting of evidence-based frailty interventions.

## Data Availability

The data analyzed in this study is subject to the following licenses/restrictions: the Cardiovascular Health Study is a community-based, prospective, observational study with data available by application to the Cardiovascular Health Study steering committee. Requests to access these datasets should be directed to https://chs-nhlbi.org.
