# Supplementary material for: Development and internal validation of an age less-dependent frailty score in the cardiovascular health study
Source: Front Med (Lausanne). 2025 Dec 11;12:1718015. doi: 10.3389/fmed.2025.1718015 (PMC12715521; doi:10.3389/fmed.2025.1718015)
Supplement: Supplementary file 1 [file Data_Sheet_1.pdf]

SUPPLEMENTAL APPENDIX

Table S1. Full List of Candidate Variables*

**Clinical Variables**†	Height, Weight, Use of anti-hypertensive medication, BP, Diabetes, Heart failure, Stroke, TIA, History of myocardial infarction, History of angina, Smoking, Peripheral arterial disease, Atrial fibrillation, History of pacemaker/ICD, Cognitive function (3MSE, DSST), Airway obstruction (FEV1)‡, Chronic kidney disease, eGFRcr-cys§, Depressive symptoms (CESD-10)‖, Alcohol intake
**Serum Biomarkers**	Lipids: LDL-C, HDL-C, Triglyceride, Lp(a). Coagulation Factors: Fibrinogen, Factor VIIc, Factor VIIIc, Platelet count. Inflammation Factors: Albumin, C-Reactive Protein, White Cells, IL-6, sCD14, sCD163, sIL-2Rα, Lp-PLA2, Galectin-3, IL-1RA, IL-18, sTNFR1. Hormonal Factors: Fasting Glucose, Fasting Insulin. Renal markers: Cystatin C, Creatinine. Cardiac markers: BNP, Troponin, ST2. Other: Uric Acid, Potassium
**Echocardiographic Variables**	E-wave velocity, A-wave velocity, E/A velocity ratio, Isovolumetric relaxation time, Left ventricular end diastolic dimension, Left ventricular end systolic dimension, Posterior wall diastolic thickness, Interventricular septal wall diastolic thickness, Relative wall thickness, Left atrial anteroposterior dimension, Aortic root dimension, Fractional shortening, LV mass, Actual to expected FS ratio, Mitral annular calcification, Aortic ring calcification, Aortic CW velocity, Aortic leaflet thickening and excursion, Tricuspid regurgitant CW velocity, Aortic valve VTI and peak velocity, LVOT VTI, Speckle tracking global longitudinal strain
**Demographic**	Age, Sex, Race¶, Hispanic origin, Level of education**, Marital status, Income‡‡

* Demographic and clinical data obtained at during the Cardiovascular Health Study baseline examination which consisted of a home interview / form and a clinic examination and took place in 1989-90 for cohort 1 and 1992-93 for cohort 2.

† Clinical history questions were obtained via self-report, with the question, “has a doctor ever told you that you had…” or “have you been told by a doctor that you currently have any of the following conditions…”?

‡ FEV1 was measured using a water-sealed spirometer.

§ Estimated glomerular filtration rate (eGFR), reported in mL/min/1.73 m^2 of body surface area, was calculated using the CKD-Epi equation using creatinine and cystatin c.

|| Measured using the Center for Epidemiologic Studies Depression (CES-D) Scale, which has a range of 0-60 based on symptoms experienced in the past week, with higher scores indicating more severe depression.

¶ Race was determined by subject self-report, with options being White, Black, American Indian/Alaskan native, Asian/Pacific Islander, and other: specify, and was included in the study to inform generalizability.

** We grouped educational attainment into 5 groups based on highest level of education, any lower-middle school, any high school, any vocational school, any college, and any graduate or professional school.

‡‡ Annual household income, self-reported using ranges, with options including <$5,000, $5,000-7,999, $8,000-11,999, $12,000-15,999, %16,000-24,999, $,25,000-$34,999, $35,000-49,999, and over $50,000, and has not been adjusted for inflation.

Table S2. Multivariate Analysis of Candidate Variables*

Continuous Variable	Number of observations	Coefficient to normalized frailty	Coefficient to normalized age	Difference of absolute coefficients (FI - Age)
Depression	3014	0.283	0.048	0.235
Glucose	2985	0.152	0.017	0.135
C-reactive protein	2982	0.125	-0.002	0.123
Income	2759	-0.227	-0.109	0.118
Interleukin-1-receptor agonist	2884	0.131	-0.018	0.113
Alcohol use	3009	-0.156	-0.051	0.105
Insulin	2970	-0.098	-0.019	0.079
White blood cells	2979	0.115	0.041	0.074
Height	3015	-0.161	-0.093	0.068
FEV1	2921	-0.266	-0.205	0.061
IL-6	2760	0.141	0.08	0.061
Fibrinogen	2982	0.114	0.059	0.055
sCD14	2794	0.076	0.028	0.048
sCD163	2717	0.082	0.046	0.036
Albumin	2982	-0.095	-0.061	0.034
TNF-α receptor-1	2884	0.200	0.166	0.034
Triglycerides	2992	0.058	-0.044	0.014
IL-18	2884	0.025	-0.011	0.014
Cystatin C	2716	0.210	0.197	0.013
Lipoprotein (a)	2633	-0.013	-0.001	0.012
**Categorical Variable**	**Number of observations**	**F value for frailty**	**F value for age**	**Difference of F values (FI - Age)**
Diabetes	2960	43.40	1.67	41.73
Deep vein thrombosis	2922	41.90	16.04	25.86
Male sex	3021	27.74	7.45	20.29
Hypertension	3014	26.52	8.74	17.79
Stroke	3021	16.49	3.87	12.62
Heart failure	3021	11.89	2.21	9.67
Education	3016	17.72	9.13	8.59
Emphysema	2985	8.13	0.16	7.97
Claudication	3021	7.93	0.10	7.82
Occupation	2642	8.98	3.05	5.93

*All variables assessed by interview, clinical exam, and phlebotomy during the Cardiovascular Health Study baseline examination.

Figure S1. Participant Flow Diagram

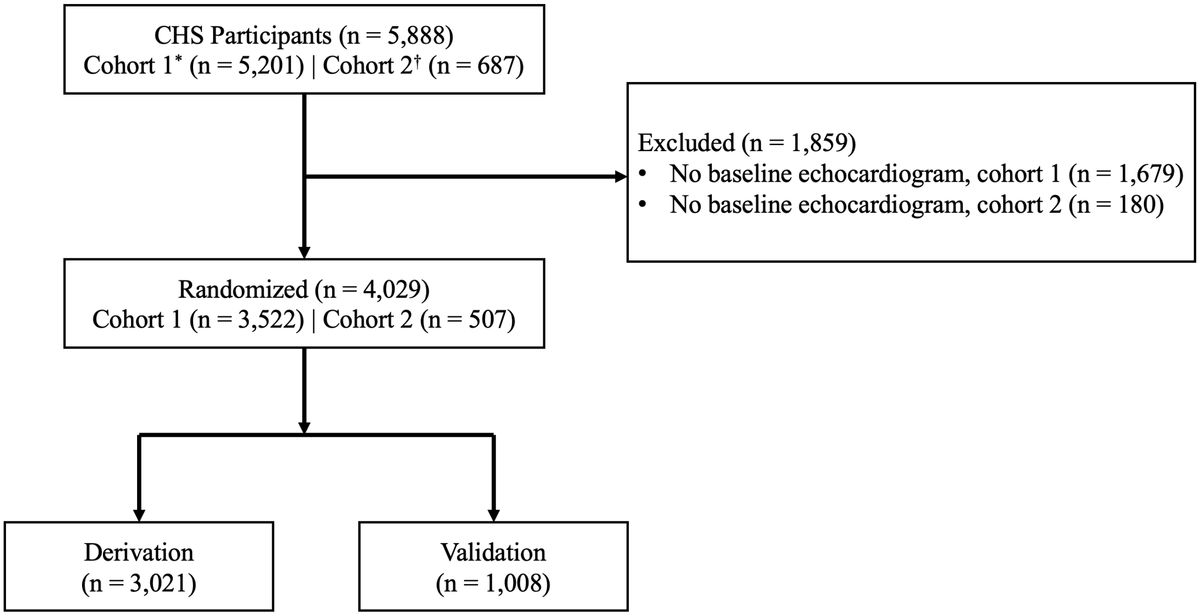

*The first Cardiovascular Health Study cohort, recruited in 1989-1990, included predominantly white individuals.

†The second Cardiovascular Health Study, recruited in 1992-1993, recruited individuals who self-identified as Black to increase diversity.

Table S3. Full Participant Characteristics

Characteristics*	Overall	Derivation (n = 3,021)	Validation (n = 1,008)	P-Value
**Demographics**				
Age (mean ± SD)	72.1±5.0	72.1±5.0	72.2±5.1	0.64
Education				0.60
Any lower-middle school	12.2%	12.5% (11.3%,13.7%)	11.5% (9.5%,13.6%)	
Any high school	41.3%	41.4% (39.7%,43.2%)	40.7% (37.7%,43.8%)	
Any vocational school	8.7%	8.6% (7.6%,9.7%)	8.9% (7.2%,10.8%)	
Any college	26.6%	26.6% (25.1%,28.2%)	26.4% (23.7%,29.2%)	
Any graduate or professional	11.3%	10.8% (9.8%,12.0%)	12.5% (10.6%,14.8%)	
Household income (mean ± SD)	5.1±2.0	5.1±2.0	5.2±1.9	0.27
Female sex	59.6%	60.0% (58.2%,61.7%)	58.3% (55.2%,61.4%)	0.36
Race				0.99
White	83.5%	83.5% (82.2%,84.9%)	83.4% (81.0%,85.7%)	
Black	16.0%	16.0% (14.7%,17.3%)	16.1% (13.9%,18.5%)	
Other	0.5%	0.5% (0.3%,0.8%)	0.5% (0.2%,1.2%)	
Marital status				0.62
Married	67.9%	68.0% (66.3%,69.7%)	67.3% (64.3%,70.2%)	
Widowed	23.2%	22.7% (21.2%,24.3%)	24.5% (21.9%,27.3%)	
Divorced	3.9%	4.1% (3.4%,4.9%)	3.3% (2.3%,4.6%)	
Separated	1.1%	1.1% (0.7%,1.5%)	1.1% (0.5%,1.9%)	
Never Married	4.0%	4.1% (3.4%,4.8%)	3.8% (2.7%,5.1%)	
Occupation (1989-90)				0.86
Professional/Technical/Managerial/Admin.	37.9%	38.1% (36.3%,40.0%)	37.1% (33.9%,40.4%)	
Sales/Clerical Services	15.6%	15.4% (14.0%,16.8%)	16.3% (13.9%,18.9%)	
Craftsman/Machine Operator/Laborer	13.8%	13.5% (12.2%,14.9%)	14.6% (12.3%,17.1%)	
Farming/Forestry	1.8%	1.7% (1.3%,2.3%)	1.9% (1.1%,3.1%)	
Homemaking	22.6%	23.0% (21.4%,24.7%)	21.6% (18.9%,24.4%)	
Other	8.3%	8.2% (7.2%,9.3%)	8.6% (6.8%,10.6%)	
Occupation (1994-95)				0.32
Employed full- or part-time	6.5%	6.8% (5.9%,7.7%)	5.8% (4.4%,7.4%)	
Homemaking	23.4%	23.7% (22.2%,25.3%)	22.4% (19.9%,25.1%)	
Employed, temporarily away	0.2%	0.3% (0.1%,0.5%)	0.1% (0.0%,0.6%	
Retired	49.6%	49.6% (47.8%,51.4%)	49.5% (46.3%,52.6%)	
Retired and working for pay	4.6%	4.3% (3.6%,5.1%)	5.5% (4.2%,7.1%)	
Retired and volunteering	12.5%	12.0% (10.9%,13.3%)	13.8% (11.7%,16.0%)	
Unemployed	1.3%	1.5% (1.1%,2.0%)	1.0% (0.5%,1.8%)	
Other	1.8%	1.8% (1.4%,2.3%)	2.0% (1.2%,3.1%)	
Height, cm (mean ± SD)	165.0±9.4	164.8±9.4 (164.5,165.1)	165.5±9.3 (164.9,166.1)	0.04
Weight, lbs (mean ± SD)	160.7±31.2	160.7±31.1 (159.6,161.9)	160.8±31.4 (158.8,162.7)	0.97

**Clinical History**				
Angina	15.0%	15.2% (13.9%,16.5%)	14.4% (12.3%,16.7%)	0.53
Bronchitis	20.3%	20.6% (19.2%,22.1%)	19.2% (16.8%,21.8%)	0.33
Cancer	18.6%	18.8% (17.4%,20.2%)	18.2% (15.8%,20.7%)	0.65
Diabetes	13.8%	13.6% (12.4%,14.9%)	14.4% (12.3%,16.8%)	0.53
eGFR (mean ± SD)	74.7±16.7	74.8±16.9 (74.1,75.4)	74.4±16.2 (73.3,75.5)	0.56
Emphysema	3.1%	3.1% (2.5%,3.8%)	3.2% (2.2%,4.5%)	0.88
Heart failure	3.1%	3.1% (2.5%,3.8%)	3.0% (2.0%,4.2%)	0.87
Hypertension	45.1%	44.9% (43.1%,46.7%)	45.6% (42.5%,48.8%)	0.70
Myocardial infarction	7.7%	7.6% (6.7%,8.7%)	7.9% (6.3%,9.8%)	0.77
Smoking status				0.75
Never smoked	47.2%	47.5% (45.7%,49.3%)	46.2% (43.1%,49.3%)	
Current smoker	42.4%	42.1% (40.3%,43.9%)	43.4% (40.3%,46.5%)	
Former smoker	10.4%	10.4% (9.3%,11.5%)	10.4% (8.6%,12.5%)	
Stroke	3.0%	3.1% (2.6%,3.8%)	2.6% (1.7%,3.8%)	0.36
Transient Ischemic Attack	2.4%	2.4% (1.9%,3.0%)	2.5% (1.6%,3.6%)	0.86
Frailty (mean ± SD) †	-0.1±0.9	-0.1±0.9	-0.2±1.0	0.70

*All variables assessed by interview and clinical exam during the Cardiovascular Health Study baseline examination.

† Frailty is measured using the continuous frailty score created by Wu, et al. based on the Fried frailty phenotype, comprised of gait speed, grip strength, exhaustion, physical activity, and weight loss, with a population mean of 0.

Table S4. Age-Stratified Association between AGELESS Score, Fried Phenotype, and Mortality

	AGELESS Score HR (95% CI)	AGELESS Score p-value	Fried Phenotype HR (95% CI)	Fried Phenotype p-value
**All-Cause Death**				
**Age quartile 1 (65-68 years)**				
AGELESS quartile 2 vs. quartile 1	0.85 (0.55-1.32)	0.48	0.91 (0.60-1.38)	0.66
AGELESS quartile 3 vs. quartile 1	1.02 (0.66-1.57)	0.93	1.06 (0.70-1.61)	0.77
AGELESS quartile 4 vs. quartile 1	1.65 (1.09-2.51)	0.02	1.55 (1.03 ,2.34)	0.03
**Age quartile 2 (68-71 years)**				
AGELESS quartile 2 vs. quartile 1	1.15 (0.70-1.90)	0.58	1.18 (0.73-1.89)	0.51
AGELESS quartile 3 vs. quartile 1	1.48 (0.91-2.42)	0.11	1.52 (0.95-2.41)	0.078
AGELESS quartile 4 vs. quartile 1	1.39 (0.84-2.31)	0.20	1.74 (1.09-2.79)	0.02
**Age quartile 3 (71-75 years)**				
AGELESS quartile 2 vs. quartile 1	1.00 (0.69-1.45)	0.98	1.03 (0.72-1.47)	0.88
AGELESS quartile 3 vs. quartile 1	1.15 (0.79-1.68)	0.46	1.01 (0.71-1.43)	0.97
AGELESS quartile 4 vs. quartile 1	1.70 (1.16-2.47)	<0.01	1.75 (1.23-2.48)	<0.01
**Age quartile 4 (75-92 years)**				
AGELESS quartile 2 vs. quartile 1	0.93 (0.61-1.42)	0.74	1.12 (0.76-1.67)	0.57
AGELESS quartile 3 vs. quartile 1	1.29 (0.85-1.95)	0.24	1.44 (0.97-2.14)	0.07
AGELESS quartile 4 vs. quartile 1	1.53 (1.00-2.33)	0.049	1.91 (1.28-2.85)	<0.01
**AIC* for All-Cause Death**	7250.08		7250.08	
**Cardiovascular Death**				
**Age quartile 1 (65-68 years)**				
AGELESS quartile 2 vs. quartile 1	0.49 (0.22-1.12)	0.09	0.78 (0.35-1.73)	0.54
AGELESS quartile 3 vs. quartile 1	0.76 (0.36-1.62)	0.48	1.11 (0.52-2.36)	0.79
AGELESS quartile 4 vs. quartile 1	1.49 (0.75-2.94)	0.26	1.47 (0.69-3.13)	0.32

**Age quartile 2 (68-71 years)**				
AGELESS quartile 2 vs. quartile 1	1.44 (0.64-3.24)	0.38	2.93 (1.15-7.49)	0.02
AGELESS quartile 3 vs. quartile 1	0.84 (0.32-2.21)	0.73	2.52 (0.94-6.73)	0.07
AGELESS quartile 4 vs. quartile 1	1.46 (0.62-3.45)	0.39	2.55 (0.93-7.03)	0.07
**Age quartile 3 (71-75 years)**				
AGELESS quartile 2 vs. quartile 1	0.64 (0.29-1.43)	0.27	1.25 (0.69-2.28)	0.47
AGELESS quartile 3 vs. quartile 1	1.27 (0.63-2.57)	0.50	1.08 (0.58-1.99)	0.81
AGELESS quartile 4 vs. quartile 1	2.68 (1.42-5.06)	<0.01	1.93 (1.06-3.50)	0.03
**Age quartile 4 (75-92 years)**				
AGELESS quartile 2 vs. quartile 1	0.64 (0.30-1.35)	0.24	0.75 (0.38-1.49)	0.42
AGELESS quartile 3 vs. quartile 1	1.53 (0.81-2.92)	0.19	1.58 (0.86-2.89)	0.14
AGELESS quartile 4 vs. quartile 1	1.53 (0.77-3.05)	0.23	1.75 (0.92-3.30)	0.09
**AIC† for Cardiovascular Death**	2116.27		2455.83	

* AGELESS Score reported by quartile, from lowest (0) to highest (3).

† AIC = Akaike information criterion, a measure of model predictive fit, with lower AIC indicating better fit.

Figure S2. AGELESS Score and Cardiovascular Disease Outcomes

A. Myocardial infarction

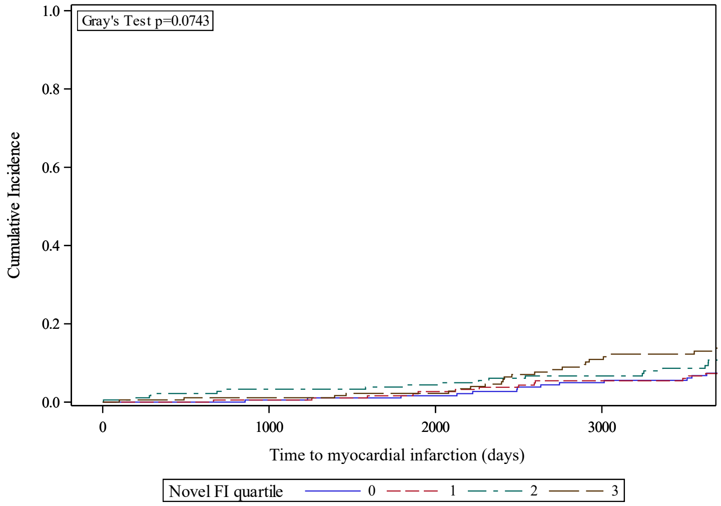

B. Heart Failure

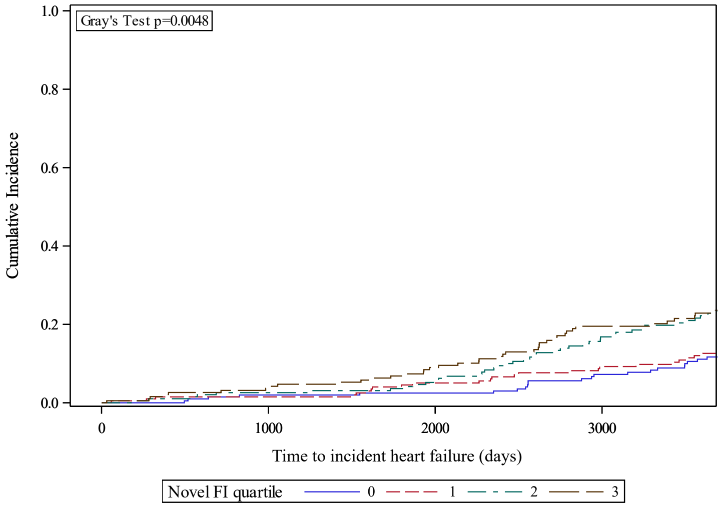

C. Stroke or Transient Ischemic Attack

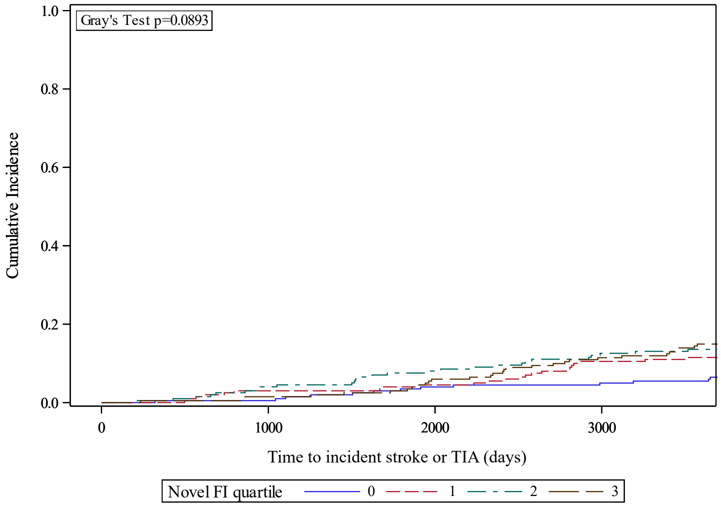

*AGELESS Score reported by quartile, from lowest (0) to highest (3).

Table S5. AGELESS Score and Cardiovascular Disease Outcomes with and without Adjusting for Age

Myocardial infarction, adjusted for age	HR (95% CI)	p-value
AGELESS quartile 2 vs. 1*	1.07 (0.66-1.75)	0.77
AGELESS quartile 3 vs. 1	1.20 (0.74-1.95)	0.46
AGELESS quartile 4 vs. 1	1.40 (0.87-2.24)	0.17
**Myocardial infarction, unadjusted**		
AGELESS quartile 2 vs. 1	1.07 (0.66-1.74)	0.79
AGELESS quartile 3 vs. 1	1.16 (0.71-1.88)	0.56
AGELESS quartile 4 vs. 1	1.32 (0.83-2.12)	0.25
**Heart failure, adjusted**		
AGELESS quartile 2 vs. 1	1.21 (0.87-1.69)	0.25
AGELESS quartile 3 vs. 1	1.38 (0.99-1.92)	0.06
AGELESS quartile 4 vs. 1	1.27 (0.90-1.81)	0.18
**Heart failure, unadjusted**		
AGELESS quartile 2 vs. 1	1.22 (0.88-1.70)	0.23
AGELESS quartile 3 vs. 1	1.42 (1.02-1.99)	0.04
AGELESS quartile 4 vs. 1	1.36 (0.97-1.92)	0.08
**Stroke or Transient Ischemic Attack, adjusted**		
AGELESS quartile 2 vs. 1	1.54 (0.97-2.43)	0.07
AGELESS quartile 3 vs. 1	1.78 (1.13-2.79)	0.01
AGELESS quartile 4 vs. 1	1.44 (0.90-2.32)	0.13
**Stroke or Transient Ischemic Attack, unadjusted**		
AGELESS quartile 2 vs. 1	1.53 (0.97-2.43)	0.07
AGELESS quartile 3 vs. 1	1.78 (1.13-2.79)	0.01
AGELESS quartile 4 vs. 1	1.44 (0.90-2.30)	0.13

* AGELESS Score reported by quartile, from lowest (0) to highest (3).

Table S6. Correlation between AGELESS Score and its Components with Fried Frailty Phenotype Components

Predictor vs. Components	Correlation*	p-value
**AGELESS Score and frailty components**		
AGELESS and grip strength	-0.35 (-0.41, -0.28)	<0.001
AGELESS and physical activity	-0.20 (-0.27, -0.13)	<0.001
AGELESS and walk speed	-0.36 (-0.42, -0.30)	<0.001
AGELESS and weight loss	0.03 (-0.05, 0.10)	0.50
AGELESS and exhaustion	0.39 (0.33, 0.45)	<0.001
**Depression and frailty components**		
Depression and grip strength	-0.17 (-0.23, -0.11)	<0.001
Depression and physical activity	-0.10 (-0.17, -0.04)	<0.01
Depression and walk speed	-0.2 (-0.27, -0.15)	<0.001
Depression and weight loss	-0.002 (-0.07, 0.06)	0.96
Depression and exhaustion	0.41 (0.36, 0.46)	<0.001
**Income and frailty components**		
Income and grip strength	0.18 (0.12, 0.24)	<0.001
Income and physical activity	0.10 (0.04, 0.17)	<0.01
Income and walk speed	0.26 (0.20, 0.32)	<0.001
Income and weight loss	0.09 (0.02, 0.15)	<0.01
Income and exhaustion	-0.16 (-0.22, -0.09)	<0.001
**FEV1 and frailty components**		
FEV1 and grip strength	0.50 (0.44, 0.54)	<0.001
FEV1 and physical activity	0.20 (0.14, 0.26)	<0.001
FEV1 and walk speed	0.25 (0.19, 0.31)	<0.001
FEV1 and weight loss	-0.03 (-0.09, 0.04)	0.38
FEV1 and exhaustion	-0.13 (-0.19, -0.07)	<0.001
**Cystatin C and frailty components**		
Cystatin C and grip strength	0.01 (-0.06, 0.07)	0.84
Cystatin C and physical activity	-0.08 (-0.14, -0.01)	0.02
Cystatin C and walk speed	-0.18 (-0.25, -0.12)	<0.001
Cystatin C and weight loss	0.10 (0.03, 0.16)	<0.01
Cystatin C and exhaustion	0.11 (0.04, 0.17)	<0.01
**Diabetes and frailty components**		
Diabetes and grip strength	0.03 (-0.03, 0.10)	0.34
Diabetes and physical activity	0.002 (-0.06, 0.07)	0.96
Diabetes and walk speed	-0.08 (-0.14, -0.02)	0.01
Diabetes and weight loss	0.01 (-0.05, 0.08)	0.73
Diabetes and exhaustion	0.08 (0.01, 0.14)	0.02
**Education and frailty components**		
Education and grip strength	0.07 (0.01, 0.14)	0.03
Education and physical activity	0.08 (0.01, 0.14)	0.02
Education and walk speed	0.19 (0.13, 0.25)	<0.001
Education and weight loss	0.03 (-0.03, 0.09)	0.36
Education and exhaustion	-0.08 (-0.14, -0.02)	0.01

* Pearson correlation coefficient (95% confidence interval)
